# Immunoglobulins and serum proteins impair anti-tumor NK cell effector functions in malignant ascites

**DOI:** 10.3389/fimmu.2024.1360615

**Published:** 2024-04-05

**Authors:** Antonio Hrvat, Sonja Benders, Rainer Kimmig, Sven Brandau, Nina Mallmann-Gottschalk

**Affiliations:** ^1^ Experimental and Translational Research, Department of Otorhinolaryngology, University Hospital Essen, Essen, Germany; ^2^ Department for Trauma Surgery and Orthopedics, St. Joseph Hospital Kupferdreh, Essen, Germany; ^3^ Department of Gynecology and Obstetrics, University Hospital Essen, Essen, Germany; ^4^ German Cancer Consortium, Partner Site Essen-Düsseldorf, Essen, Germany; ^5^ Department of Gynecology and Obstetrics, University Hospital Cologne, Cologne, Germany

**Keywords:** NK cells, ascites, immunosuppression, ovarian cancer, tumor microenvironment, albumin, antibody, immunoglobulins

## Abstract

**Introduction:**

Malignant ascites indicates ovarian cancer progression and predicts poor clinical outcome. Various ascites components induce an immunosuppressive crosstalk between tumor and immune cells, which is poorly understood. In our previous study, imbalanced electrolytes, particularly high sodium content in malignant ascites, have been identified as a main immunosuppressive mechanism that impaired NK and T-cell activity.

**Methods:**

In the present study, we explored the role of high concentrations of ascites proteins and immunoglobulins on antitumoral NK effector functions. To this end, a coculture system consisting of healthy donor NK cells and ovarian cancer cells was used. The anti-EGFR antibody Cetuximab was added to induce antibody-dependent cellular cytotoxicity (ADCC). NK activity was assessed in the presence of different patient ascites samples and immunoglobulins that were isolated from ascites.

**Results:**

Overall high protein concentration in ascites impaired NK cell degranulation, conjugation to tumor cells, and intracellular calcium signaling. Immunoglobulins isolated from ascites samples competitively interfered with NK ADCC and inhibited the conjugation to target cells. Furthermore, downregulation of regulatory surface markers CD16 and DNAM-1 on NK cells was prevented by ascites-derived immunoglobulins during NK cell activation.

**Conclusion:**

Our data show that high protein concentrations in biological fluids are able to suppress antitumoral activity of NK cells independent from the mechanism mediated by imbalanced electrolytes. The competitive interference between immunoglobulins of ascites and specific therapeutic antibodies could diminish the efficacy of antibody-based therapies and should be considered in antibody-based immunotherapies.

## 1 Introduction

Malignant ascites indicates the development of peritoneal carcinomatosis in advanced or recurrent stages of epithelial ovarian cancer, especially in high-grade serous carcinomas (HGSC) (1). This peritoneal accumulation of fluid deteriorates the patients´ physical state, limits their survival (2), and is connected to aggressive tumor biology and chemoresistance (3, 4). There is clear evidence that both host immune cells and soluble ascites components substantially contribute to a highly proinflammatory and immunosuppressive tumor microenvironment that promotes disease progression (5–7). Since the complex interactions between ascites, cancer cells, and infiltrated immune cells are still poorly understood, the efficacy of systemic and novel immune-based therapies is reduced (8, 9).

Next to suppressive immune cells (6), acellular ascites components may also downregulate immune responses in ascites. Several immunoregulatory proteins such as cytokines, soluble growth factors, integrins, and shedded tumor ligands promote proinflammatory environment (10–14). For example, IL-6 was reported to impair NK cell effector functions and support ovarian tumor growth *in vivo* (15, 16). Anti-inflammatory cytokine IL-10 similarly suppresses T-cell activation (17). There are also many reports showing that released ligands such as MICA, MICB, or ULBP-2 can block NKG2D receptors on NK cells and CD8+ T cells and impair antitumor function (18, 19). Lastly, besides these classical suppressive molecules, even acute-phase proteins and immunoglobulins in serum were found to impair immune effector function (20–22).

Since previous attempts to characterize soluble inhibitory factors in ascites led to inconsistent results (23), in a recent study, we explored the biology and mechanism of potential immunosuppressors in malignant ascites. For this, we obtained ascites samples from patients with advanced or recurrent ovarian cancer and depleted their cellular components. In an *in vitro* coculture model consisting of healthy donor T and NK cells, EGFR-positive cancer cells, and ADCC (antibody-dependent cellular cytotoxicity)-inducing anti-EGFR-antibody Cetuximab, we studied different antitumoral effector functions in the presence of acellular ascitic fluid. Using this approach, we identified imbalanced electrolytes as a novel mechanism and main source of immunosuppression in malignant ascites (24). However, functional experiments suggested that electrolyte imbalance only partially explained suppression, which implies the existence of additional mechanisms.

Therefore, using our established *in vitro* coculture model, we incubated NK cells in the ascitic fluid and correlated their activity with the protein content of the samples. Furthermore, we also studied the immunosuppressive potential of antibodies contained in malignant ascites. This study provides insight into the immunoregulatory role of proteins and immunoglobulins, which act independently from imbalanced electrolytes in malignant ascites.

## 2 Materials and methods

### 2.1 Patient ascites samples, patient and healthy donor serum preparation

**Figure 1:**
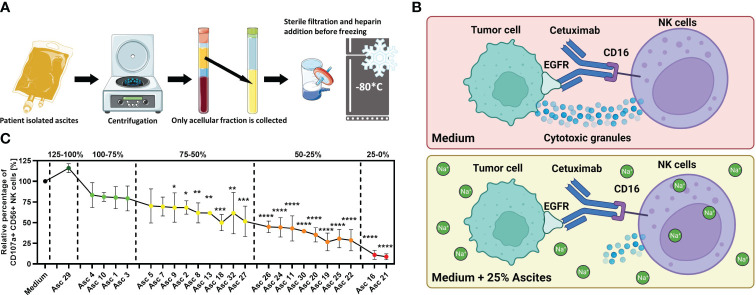
Malignant peritoneal ascites impairs NK cell effector functions *in vitro*. **(A)** Processing of ascites samples. Graphical illustration of initial processing of ascites samples derived from patients with malignant ascites and storage of ascitic fluid after depletion of cellular components. **(B)** Illustration of the NK–tumor cell coculture system under the ADCC condition. This experimental setup was used to assess the effects of ascites or healthy donor serum on NK cell effector function. The same setup in the previous study was used, which revealed that malignant ascites is a sodium-imbalanced environment (24). Illustrations were created with BioRender and Sevier Medical Art. **(C)** Antibody-dependent cellular cytotoxicity (ADCC) of NK cells (NK) in the presence of selected ascites samples. Resting healthy donor NK cells were coincubated in 1:1 ratio with IGROV1- cells for 6 h with the addition of ADCC-inducing anti-EGFR-antibody Cetuximab (1 µg/mL) and benign ascites (Asc 29) or malignant ascites (all other 23 samples). We could differentiate samples with only weak (green), with intermediate (yellow), strong (orange), and very strong (red) inhibitory potential and sample no. 29 with no inhibition of NK ADCC (dark green) (24). Each datapoint represents one healthy NK cell donor. The relative percentages are shown after normalization to normal medium control. Data are presented as individual values with mean value as center of error bar ± standard deviation. Each datapoint represents one healthy donor. The normalization was done according to normal medium control. For significance testing, ordinary one-way **(C)** ANOVA and Dunnett’s post-hoc test were used.

Twenty-eight patient ascites samples with advanced malignancies were collected at the Departments of Gynecology and Obstetrics at the University Hospital Cologne, Cologne and the University Hospital Essen, Essen, Germany. Among them, 14 patients with initial and 6 patients with recurrent diagnosis of high-grade serous ovarian carcinoma, and 2 patients with initial diagnosis of invasive mucinous ovarian carcinoma, one small cell carcinoma of the ovary, hypercalcemic type (SCCOHT) and one ovarian signet ring cell carcinoma, have been enrolled. In addition, one patient with initial advanced colon carcinoma and one patient with adenocarcinoma of unknown primary have been included. Besides these, one patient with diffuse large cell B-cell lymphoma and one patient with benign ascites due to congestive heart failure were part of the cohort. The ascites samples were collected during clinically indicated surgery or palliative paracentesis after written information and consent. The isolated ascites was transferred to Falcon 50 tubes and centrifuged at 2,000*g* for 10 min. After centrifugation, only acellular supernatant was collected and sterile filtered using bottle top filters (Sarstedt, 0.2 μm). Patient and healthy donor serum was obtained using clotting activator serum collection tubes (Sarstedt). Samples were centrifuged at 2,000*g* for 10 min. All samples were aliquoted and frozen at −80°C for long-term storage. The procedure is illustrated in **Figure 1A**. The study has been approved by the local Ethics Committee of the medical faculties of the Universities of Duisburg-Essen and Cologne.

### 2.2 Human cancer cell lines and *in vitro* cell culture

Several EGFR-positive cell line models were used in coculture assays: The human ovarian cancer cell line IGROV1 was kindly provided by LIMES Institute, University of Bonn, Bonn, Germany. SKOV-3 ovarian cancer cell lines were kindly provided by the Department of Obstetrics and Gynecology, University of Bonn, Germany. The colorectal cancer cell line A431 was obtained from DSMZ, Braunschweig, Germany. All used cancer cell lines showed positive expression for EGFR (epidermal growth factor receptor). IGROV1, SKOV3, and A431 cell lines were cultured in Roswell Park Memorial Institute (RPMI Gibco) supplemented with 10% (v/v) heat-inactivated fetal calf serum (FCS Gibco), 100 U/mL penicillin, and 100 mg/mL streptomycin (PenStrep, Gibco by Life Technologies) (supplemented complete media). Cell cultivation was done in plastic flasks (Sarstedt) at 37°C and 5% CO_2_ and deattached for passaging by Accutase (Gibco) treatment for 5 min at 37°C. Before use, the cell lines were tested to exclude mycoplasma contamination. Cell line identity was confirmed by STR Typing done by the Leibniz-Institute DSMZ Molecular Biology Group.

### 2.3 Isolation of healthy donor NK cells from peripheral blood

Healthy donor blood was collected in trisodium citrate blood collection tubes (Sarstedt). Blood was diluted (1:1 ratio) with Dulbecco’s Phosphate Buffered Saline (DPBS, Gibco, Life Technologies Limited) and overlaid on a separation medium (1.077 g/mL, Biocoll, Merck Millipore). Separation by density was done by centrifugation at room temperature (400*g* for 30 min) without acceleration or brake. Collected PBMCs were incubated in a T175 plastic flask (Sarstedt) at 37°C and 5% CO_2_ for 1 h for depleting monocytes. NK isolation was performed using NK MACS Isolation Kit (Miltenyi Biotec) according to the manufacturer’s instructions. After isolation, 5 U/mL recombinant human IL-15 (50 µg, Immuno Tools) was added to NK cells that were used for functional experiments after incubation overnight.

### 2.4 NK cell degranulation assay

Isolated NK cells were cocultured (1:1 ratio, 25,000 each) with ovarian cancer cells in a flat-bottom 96-well plate with or without different patient ascites or ascites-isolated antibody samples (25% addition), bovine serum albumin [fraction V (pH 7.0), PanReac], or Rituximab (MabThera, 10 mg/mL, Roche). The human anti-EGFR-antibody Cetuximab 1 μg/mL [Erbitux, 5 mg/mL, Merck (Serono)] was added to the coculture to induce NK cell-mediated ADCC. For induction of unspecific degranulation, PMA (Sigma-Aldrich, 50 ng/mL) and Ionomycin (Sigma-Aldrich, 1 µg/mL) were added. NK cells were labeled with anti-CD107a-FITC (25 µg/mL, clone H4A3, Mouse IgG1, k, BD Biosciences) or isotype control and incubated for 1 h at 37°C and 5% CO_2_. Afterwards, Golgistop Monensin (BD Biosciences) was added (1:600). Following 5 h of incubation, NK cells were stained with CD56-BV421 (12 µg/mL, NCAM 16.2, IgG2b, k, BD Biosciences). CD107a and CD56 expressions were analyzed by flow cytometry.

### 2.5 Flow cytometric analysis of NK cell markers

NK cells were coincubated in 1:1 ratio with A431 cells, Cetuximab (1 µg/mL) and 25% addition of isolated ascites antibodies for 18 h. The following antibodies for the flow cytometric analysis of the NK cell marker expression were used: CD56-BV421 (12 µg/mL, clone NCAM 16.2, mIgG2b,k, BD Biosciences), DNAM-1-PerCP-Cy5.5 (CD226, 200 µg/mL, clone 11A8, mIgG1, k, BioLegend), and CD16-PE-Cy7 (50 µg/mL, clone 3G8, mIgG1, k, BD Biosciences). Stained cells were analyzed with BD FACS Canto II using DIVA 8.01 software (BD Biosciences) or FlowJo10 (LLC, Ashland, Oregon, USA).

### 2.6 Conjugation assay

Ovarian cancer cells were prestained with Cell Tracker Green (Thermo Fisher Scientific) and NK cells were prestained with Cell Tracker Red (Thermo Fisher Scientific) according to the manufacturer’s instructions. NK cells (50,000 cells) and tumor cells (200,000 cells) were coincubated (1:4 ratio) in the presence of 1 μg/mL Cetuximab with different concentrations of albumin, Rituximab, or ascites isolated antibodies. After centrifugation [20*g* (570 rpm), 1 min], tubes were placed in a water bath at 37°C for 45 min. Retrieved samples were briefly vortexed and 300 µL of ice-cold 0.5% PFA in PBS was added. Using flow cytometry, events positive for both cell trackers were interpreted as conjugates.

### 2.7 Calcium flux assay

For the assessment of intracellular calcium, NK cells were stained with Fluo-4 (Thermo Fisher Scientific) according to the manufacturer’s specifications. Prestained NKs (250,000 cells) from original suspensions were taken and incubated in either phenol-free media and Rituximab or albumin-supplemented media in 96-well plates. This has allowed for consistent dye loading and comparison between samples. The baseline fluorescence was recorded for 1 min and then PMA (Sigma-Aldrich, 50 ng/mL) and Ionomycin (Sigma-Aldrich, 1 µg/mL) were added to induce NK cell activation. Intracellular calcium flux was measured for 10–15 min in intervals of every 3 s at 37°C using spectrophotometer Synergy2 (BioTek). Absorbance was recorded at 480/25. Data were normalized according to the average unstimulated baseline and presented as fold change of starting point measurement. Comparison between calcium flux peak values and ROC curve analysis was used to confirm significance between different measured flux curves.

### 2.8 Clinical chemistry analysis of patient ascites

Potentiometry was done to determine the concentrations of sodium, potassium, and chloride by using an ion-selective electrode on the Atellica CH Analyzer (Siemens Healthineers). Total protein (pyrogallol red method) and albumin (bromocresol green method) were photometrically determined on the Atellica CH Analyzer (Siemens Healthineers). IgG subclasses were measured by immunonephelometry on the BN-II (Siemens Healthineers). Instrument controls were performed according to the manufacturer’s instructions. Parameters are accredited according to DIN EN ISO 15189:2014. The original obtained clinical chemistry data of ascites, serum samples, and their permeates has been previously published in (24).

### 2.9 Ultracentrifugation, filtration, dialysis, and heat inactivation of ascites and healthy donor serum samples

Healthy donor serum was fractionalized using ultracentrifugation filters (Amicon, 0.5 mL, MCWO 3 kDa), which were centrifuged at 14,000–15,000*g*. The centrifugation was interrupted after 15 min and the remaining ascites was resuspended before continuing centrifugation for another 15 min. For dialysis of ascites samples, dialysis tubes were filled up with ascites and dialyzed against 1 L of cell culture medium RPMI 1640 (Pur-a-lyzer, Sigma-Aldrich, 1 kDa and 25 kDa) for 24 h. Heat inactivation of untreated or dialyzed ascites was performed at 56°C for 30 min.

### 2.10 Quantification of cytokines in ascites by ELISA

Cytokine concentrations in patient ascites samples were assessed using ELISA kits, according to the manufacturer’s instructions. The following cytokines were assessed: CXCL1, IL-6, IL-8, IL-10, IL-12, IL-13, IL-17, IL-27, MIF, MIP-1b, TGF-β1, TNF-α, and VEGF. All used ELISA kits were obtained from R&D Systems, Wiesbaden, Germany.

### 2.11 Chromatographic isolation of IgG immunoglobulins in ascites

Ascites samples were diluted (1:1 ratio) with phosphate buffer saline (pH 7.2, Thermo Scientific), and protein A/G chromatography columns (Pierce, Thermo Scientific, 1 ml volume) were equilibrated with the BupH PBS. The sample was added and pushed through the column. The column was rinsed out with binding buffer and washed with elution buffer (Thermo Scientific), which released IgG1 antibodies from the column. Neutralization buffer (100 µL; 1 M Tris, pH 9) was added per 1 mL of collected fraction. After neutralization, collected fractions were gathered and concentrated until original ascites volume was reached (500 μL). The concentration of the eluted antibodies was determined by UV spectrophotometry (OD 1.4 ≈ 1 mg/mL IgG) before *in vitro* application.

### 2.12 Statistical analysis

Plotted data depict single values, mean as center value, and error bars for the standard deviation (SD). Heatmap data points represent mean values. Performed statistical tests are described in the corresponding figure legend. All graphs and statistical tests were prepared using GraphPad Prism 8 software.

## 3 Results

### 3.1 Malignant ascites causes NK cell immunosuppression

For the initial assessment of antitumoral NK cell activity in ovarian cancer, we implemented an *in vitro* coculture system consisting of healthy donor NK cells, EGFR-positive tumor cells, and the anti-EGFR antibody Cetuximab that mediates ADCC (**Figure 1B**). In a further development, we studied different NK cell effector functions in the microenvironment of malignant ascites. For this, we supplemented our coculture with patient-derived malignant ascites after depletion of cellular components and additionally included one benign ascites derived from a patient with congestive heart failure in our study. Using this approach, we could demonstrate that crucial cytotoxic and secretory NK effector functions were substantially impaired by most of the malignant samples but not by the benign ascites sample. According to their inhibitory potential on NK ADCC, we were able to categorize all 23 ascites samples in very strong, strong, intermediate, and weak inhibitory samples and stimulatory sample (**Figure 1C**). To further study the underlying immunosuppressive mechanisms, we selected four malignant ascites samples with very strong and strong inhibitory potential (nos. 16, 21, 24, and 25) and the benign sample (no. 29) (24).

### 3.2 Malignant ascites contains additional inhibitory factors besides imbalanced electrolytes

When we analyzed the content of ascites, we identified increased sodium concentration as the primary cause of immunosuppression. Using sodium channel blockers such as amiloride or lidocaine, we could confirm this inhibitory mechanism. However, their addition resulted in partial restoration of effector function (24), which indicated another potential inhibitory factor.

**Figure 2:**
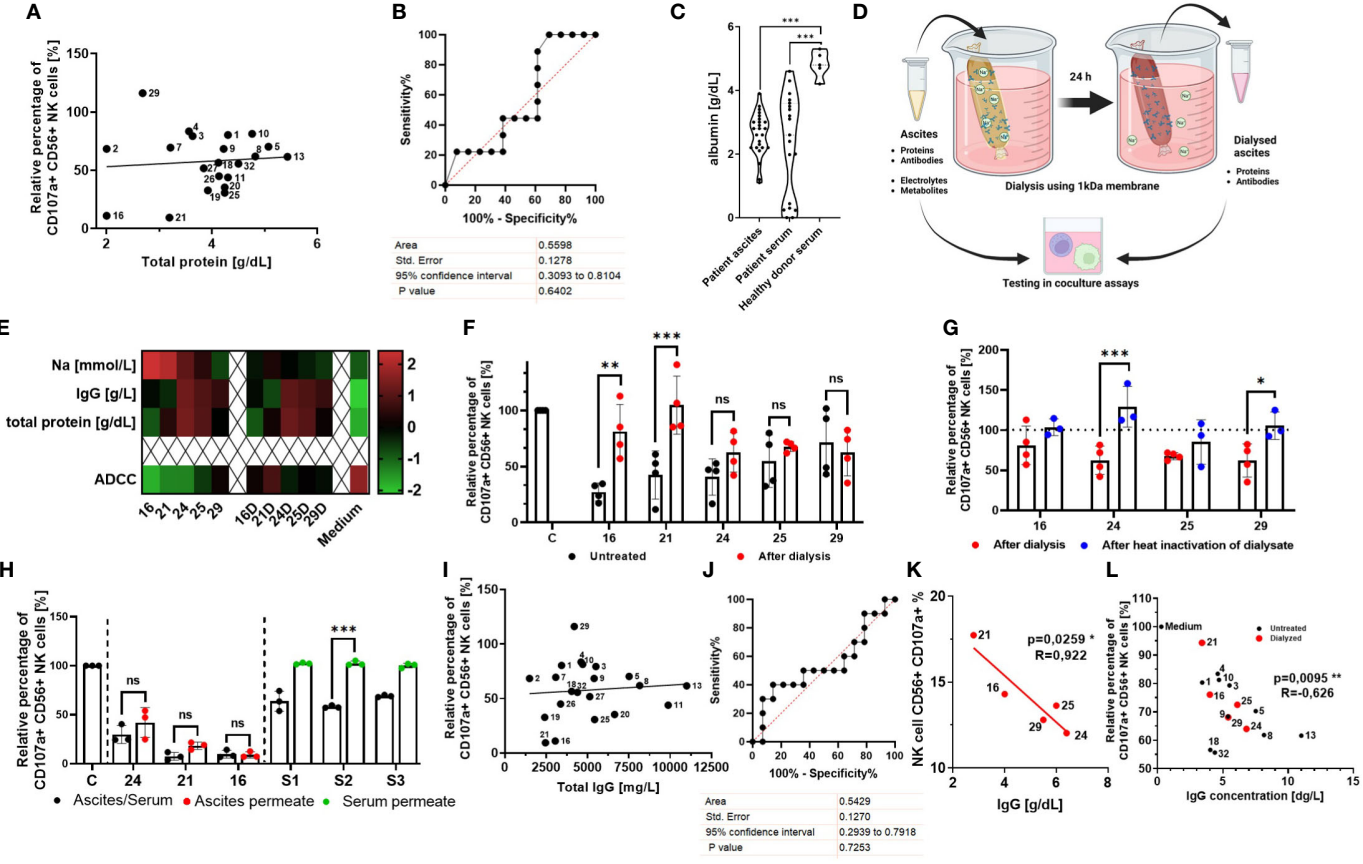
Normalization of imbalanced electrolytes in malignant ascites only partially rescues NK cell ADCC, indicating the existence of an additional inhibitory mechanism. **(A, B)** Relationship between NK ADCC and protein content in ascites samples. **(A)** Pearson correlation shows no significant correlation between NK ADCC [percentage of CD107a-positive NK cells] and protein content [g/dL]. **(B)** ROC (receiver operating characteristic) curve depicting overall protein content as nonsignificant random classifiers. **(C)** Comparison of albumin content in biological fluids. Violin plot illustrating the comparison of albumin content [g/dL] in patient ascites, patient serum, and healthy donor serum. **(D)** Dialysis of ascites samples. Schematic illustration of normalizing ascites electrolyte content via dialysis. Ascites samples were processed in medium overnight using 1-kDa cutoff dialysis tubing to normalize electrolyte composition. Inhibitory effects were assessed in coculture assays. **(E)** Ascites composition before and after dialysis. Heatmap showing concentrations of sodium, IgG, and total protein in selected ascites samples (16, 21, 24, 25 and 26) before (left) and after dialysis (right) (16D,21D,24D,25D,29D) and in culture medium for control (24). **(F)** NK ADCC in the presence of ascites before and after dialysis. Resting NK cells were coincubated in 1:1 ratio with IGROV1 cells and Cetuximab in the presence of untreated (black) or dialyzed ascites (red). After 6 h, expression of CD107a on NK cells was determined by flow cytometry (24). **(G)** NK ADCC in the presence of dialyzed ascites before and after heat inactivation. Resting NK cells were coincubated in 1:1 ratio with IGROV1-cells and Cetuximab with dialyzed ascites (red) and after additional heat inactivation at 56°C for 30 min (blue). **(H)** NK ADCC in the presence of ascites or healthy donor serum after protein depletion. Resting NK cells were coincubated in 1:1 ratio with IGROV1 cells with the addition of Cetuximab in the presence of untreated serum or ascites (black), protein-less ascites permeate (red) and serum permeate (green). After 6 h, expression of CD107a on NK cells was determined by flow cytometry. **(I, J)** Relationship between NK ADCC and IgG-concentration in ascites samples. **(I)** Pearson correlation shows no significant correlation of NK ADCC [percentage of CD107a-positive NK cells] to content of IgG-immunoglobulins [mg/L]. **(J)** ROC (receiver operating characteristic) curve depicting immunoglobulins as nonsignificant random classifiers. **(K, L)** Relationship between NK ADCC and IgG-concentrations in ascites samples with normalized or low sodium content. **(K)** Pearson correlation shows significant correlation of NK ADCC to content of IgG-immunoglobulins [mg/L] in dialyzed ascites samples (red dots). **(L)** Pearson correlation shows significant correlation of NK ADCC to IgG immunoglobulins in dialyzed ascites samples (red dots) and untreated samples with physiological or low sodium content (<145 mM) (black dots). Data are presented as individual values with mean value as center of error bar ± standard deviation. Each datapoint represents one healthy donor. The normalization was done according to normal medium control. For significance testing, ordinary one-way **(C, F, G, H)** ANOVA and Sidak’s post-hoc test, two-tailed Pearson correlation **(A, I, K, L)**, and ROC analysis **(B, J)** were used. *p < 0.05, **p < 0.01, ***p < 0.001.

Considering the heterogeneous composition and the variety of proteins contained in ascites, we first assessed whether any correlations between overall protein content and suppressive activity existed. However, Pearson correlation (**Figure 2A**) and ROC analysis (**Figure 2B**) did not reveal any significant connection to NK cell ADCC inhibition. Furthermore, we observed that healthy donor serum contained higher albumin concentrations compared to patient serum and ascites (**Figure 2C**). In conjunction with our published data, these findings suggest that contribution of the protein component to the overall inhibition is lesser than electrolyte imbalance.

In order to further assess the inhibitory potential of the ascites proteinaceous component, we normalized electrolyte content in ascites samples with high and low protein content (**Figure 2D**). Using nephelometry and potentiometry, we confirmed that dialysis restored electrolyte levels to the level of culture medium, while ascites protein and antibody content remained unchanged (**Figure 2E**) (24). In selected ascites samples with high sodium (nos. 16, 21, and 24) and low sodium content (nos. 25 and 29), NK ADCC was determined before and after dialysis (**Figure 2F**). In line with our hypothesis, the normalization of imbalanced electrolytes leads to a significant restoration of NK ADCC in samples with previously high sodium content (nos. 16, 21, and 24). In contrast, NK ADCC in the presence of ascites with previously lower sodium content (nos. 25 and 29, but also no. 24) was only slightly restored by dialysis (24). These data indicate the existence of another, additional suppressive component and mechanism that becomes more relevant under conditions of no or moderate electrolyte imbalance.

To further substantiate this hypothesis, we assessed NK ADCC in the presence of heat-inactivated ascites samples. Indeed, heat inactivation restored NK ADCC only in the presence of samples with low sodium content (**Supplementary Figure 1A**, nos. 9 and 20) while inhibitory property of sample no. 21 with high sodium content remained unaffected from heat inactivation. Furthermore, when already dialyzed ascites samples were heat inactivated, we could observe a further restoration of NK ADCC in ascites no. 24 and no. 29 (**Figure 2G**), supporting the hypothesis that the potential additional inhibitory factors are heat sensitive.

In the next series of experiments, we aimed to elucidate the nature of these potential inhibitory components. For this purpose, proteins in the original ascites samples were depleted using 3 kDa ultracentrifugation obtaining protein depleted permeate fraction, which contained only electrolytes and small metabolites. We could show that permeates of ascites samples with high sodium content remained inhibitory to the same extent as the original untreated sample (**Figure 2H**, red) (24). Following this, we assessed NK cell ADCC in the presence of healthy donor blood serum and its protein less permeate (**Figure 2H**, green). While untreated healthy donor serum exerted inhibition, no inhibition could be observed after depleting proteins. In summary, these results support the hypothesis that heat-sensitive proteins are another immunosuppressive mediator in ascites, particularly mediating immunosuppression in an electrolyte balanced environment with physiological sodium content.

Since malignant ascites is a complex tumor microenvironment containing various proinflammatory and immunosuppressive cytokines, in the following part of our study, we assessed their impact on NK effector function. First, we quantified various cytokines in 21 ascites samples using ELISA (**Supplementary Figure 1B**) and correlated their content to NK effector function (**Supplementary Figure 1C**). Among these cytokines, IL-8 exhibited significant positive correlation to NK cell ADCC (**Supplementary Figure 1D**), while IL-10 showed an inverse correlation in trend (**Supplementary Figure 1E**). However, supplementing cell culture medium with corresponding concentrations of IL-8 or IL-10 in our experimental settings had no impact on NK ADCC (**Supplementary Figure 1F**). As in our samples (**Supplementary Figures 1G, H**), malignant ascites usually contains high IL-6 and VEGF levels, which are also of prognostic relevance (12, 25). However, their addition to our experimental settings also did not have any impact on NK ADCC (data not shown).

Since ascites-related cytokines did not seem to be main regulators of cytotoxic NK effector function in our present study, we re-analyzed our original nephelometric data of ascites content. Here, we noticed that all ascites samples considerably contained IgG immunoglobulins. However, IgG content of all original ascites samples did not correlate to NK ADCC (**Figures 2I, J**). Interestingly, after analyzing the IgG content of dialyzed ascites samples with balanced electrolytes, we could reveal that IgG content was significantly inversely correlated to NK ADCC (**Figure 2K**). Importantly, not only in dialyzed samples, but also in all samples with physiological or lower sodium content (<145 mM) was IgG concentration significantly negatively correlated to NK ADCC (**Figure 2L**).

The presented data of this part of the study indicate that ascites-related proteins and IgG immunoglobulins substantially contribute to ascites-mediated immunosuppression, which is masked in a high-sodium environment but becomes evident in an environment with physiological sodium content.

### 3.3 Irrelevant antibodies and high protein content inhibit NK cell function *in vitro*

**Figure 3:**
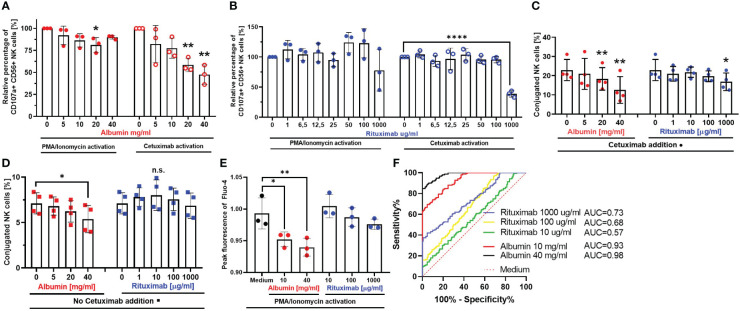
Irrelevant antibodies and serum proteins interfere with various NK cell effector functions. Spontaneous NK cell degranulation and ADCC in the presence of serum protein albumin **(A)** or irrelevant antibody Rituximab **(B)** NK cells were stimulated with PMA (50 ng/mL)/Ionomycin (1 µg/mL) (left panels) or coincubated with IGROV-1 (1:1 ratio) and Cetuximab (1 µg/mL) (right panels) in the presence of either albumin (5, 10, 20, and 40 mg/mL) or Rituximab (1, 6.5, 12.5, 25, 50, 100, and 1,000 μg/mL) for 6 h. Percentage of CD107a-positive NK cells was assessed by flow cytometry. **(C, D)** NK-TC conjugation in the presence **(C)** or absence **(D)** of Cetuximab. NK cells and IGROV1- cells were mixed in 4:1 effector-to-target ratio and the addition of albumin (5, 10, 20, and 40 mg/mL) or Rituximab (1, 10, 100, and 1,000 μg/mL) with **(C)** or without **(D)** Cetuximab (1 µg/mL). Percentage of NK-TC-conjugates was assessed by flow cytometry. **(E, F)** Intracellular calcium-flux during NK cell stimulation in the presence of albumin and Rituximab. NK cells were exposed to PMA (50 ng/mL)/Ionomycin (1 µg/mL) in the presence of either medium or medium supplemented with albumin (10 and 40 mg/mL) and Rituximab (10,100, and 1,000 μg/mL), respectively. Intracellular Ca2+ flux was monitored via Fluo-4 dye. **(E)** Fold increase of 480/25 absorbance at peak of calcium flux. **(F)** ROC analysis shows significant difference between calcium flux curves in albumin (red and black line) compared to medium (dotted red line). Each datapoint represents one ascites sample. For significance testing, ordinary one-way ANOVA **(A–E)** with Dunnett’s post-hoc test and ROC analysis **(F)** were performed. ns, non-significant; *p < 0.05, **p < 0.01, ****p < 0.0001.

In the next part of our study, we aimed to investigate whether the inhibitory mechanism of immunoglobulins is distinct from immunosuppression mediated by overall high protein content. For this purpose, we added albumin to our *in vitro* coculture, which is the most abundant protein in serum and ascites. Furthermore, we used Rituximab as an irrelevant antibody without specific target and ADCC function in our experimental setting, because CD20, the target antigen of Rituximab, is not expressed on the cell lines used in our model. Firstly, we added different concentrations of albumin (**Figure 3A**) or Rituximab (**Figure 3B**) to the coculture and assessed NK cell degranulation under target-independent conditions in the absence of tumor cells (PMA/Ionomycin activation, left panels, **Figures 3A, B**) and under ADCC conditions in the presence of Cetuximab and EGFR-positive tumor cells (right panels, **Figures 3A, B**), respectively. Our results show that albumin had the capacity to inhibit NK cell degranulation in a dose-dependent manner in both ADCC- and target-independent conditions (**Figure 3A**). In contrast, Rituximab only impaired NK cell degranulation in the presence of Cetuximab in high dosage (1 mg/mL) (**Figure 3B**).

In further experiments, we investigated the impact of albumin and Rituximab on NK–tumor cell conjugation either in the presence (**Figure 3C**) or in the absence (**Figure 3D**) of Cetuximab. According to our data, the addition of albumin caused significant dose-dependent inhibition of NK–tumor cell conjugation in both conditions (left panels, **Figures 3C, D**). According to the inhibition of NK ADCC, Rituximab only inhibited conjugation in the presence of Cetuximab in high dosage (right panel, **Figures 3C, D**.

Since the influx of calcium is an important step in early activation of immune cells, we studied the impact of different concentrations of albumin and Rituximab on the influx of calcium in NK cells during their activation by PMA and Ionomycin. We could demonstrate that calcium influx was significantly inhibited by the addition of albumin, but not in the presence of Rituximab (**Figures 3E, F**).

In summary, these mechanistic studies indicate that proteins like albumin are able to reduce cytotoxic NK cell activity and impair NK–tumor cell conjugation by inhibiting influx of calcium in NK cells. Importantly, the inhibition was independent of the presence of ADCC-inducing antibody. In contrast, irrelevant antibodies inhibited NK cytotoxicity and conjugation only in the presence of Cetuximab, while the influx of calcium in NK cells remained unaffected. The irrelevant antibodies in our system are supposed to act as competitive inhibitors in the presence of specific targeted antibody, which is distinct from the mechanism of inhibition meditated by proteins and imbalanced electrolytes.

### 3.4 Ascites IgG immunoglobulins competitively inhibit Cetuximab-induced NK cell activation

**Figure 4:**
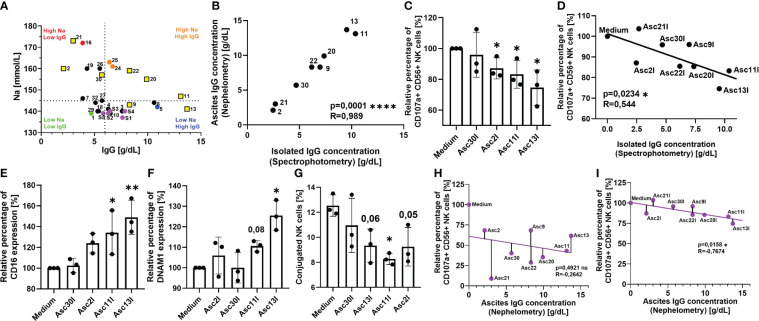
Antibodies isolated from ascites samples competitively inhibit Cetuximab-mediated NK cell cytotoxicity. **(A)** Ascites samples have heterogeneous sodium and IgG composition. Pearson correlation of sodium and IgG content in ascites and serum samples as determined by clinical chemistry. Dotted line divides ascites samples into high-sodium/low-sodium (red/orange versus green/blue) and high-IgG/low-IgG (orange/blue versus red/green) using physiological referent values for both components, serum samples are marked in purple. Yellow squares mark selected ascites samples for IgG isolation. **(B)** Quantification of ascites IgG immunoglobulins isolated by chromatography. UV absorbance at 280 nm was used to estimate amount of isolated IgG (black dots). Pearson correlation of isolated IgG immunoglobulins to nephelometry quantified ascites immunoglobulins. **(C**, **D)** NK ADCC in the presence of ascites isolated immunoglobulins. Resting healthy donor NK cells were coincubated in 1:1 ratio with A431 cells for 6 h with the addition of ADCC-inducing anti-EGFR-antibody Cetuximab (1 µg/mL) and 25% addition of isolated ascites immunoglobulins. **(C)** NK ADCC in the presence of ascites isolated immunoglobulins. NK ADCC was quantified by flow cytometry after 6 h of coincubation. **(D)** Pearson correlation shows significant correlation of NK ADCC to ascites isolated immunoglobulins. **(E, F)** Expression of surface markers on NK cells in the presence of isolated ascites immunoglobulins. Isolated healthy donor NK cells were coincubated with A431 cells (1:1 ratio) and Cetuximab (1 μg/mL) in either medium or medium supplemented with 25% of isolated immunoglobulin suspension. After 18 h, expression of **(E)** CD16 and **(F)** DNAM-1 were measured by FACS. **(G)** Ascites isolated immunoglobulins impair NK-TC conjugation. NK cells and A431 were mixed in 1:4 effector-to-target ratio in either normal medium or medium with 25% addition of isolated ascites immunoglobulins. Percentage of NK-TC-conjugates was assessed by flow cytometry. **(H, I)** Electrolyte imbalance in malignant ascites masks the inhibitory effect of immunoglobulins. **(H)** Pearson correlation of ascites IgG content to NK ADCC in the presence of unprocessed ascites. **(I)** Pearson correlation of ascites IgG content to NK ADCC in the presence of isolated ascites immunoglobulins. Each datapoint represents one healthy donor. The relative percentages are shown after normalization to normal medium control. For significance testing, two-tailed Pearson correlation **(A, B, D, H, I)**, ordinary one-way ANOVA followed by Dunnett’s post-hoc test **(E–G)**, and unpaired *t*-test **(C)** were used. ns, non-significant; *p < 0.05, **p < 0.01, ****p < 0.0001.

Since we have demonstrated that serum and ascites proteins and irrelevant antibodies can mediate inhibition, in the last part of our study, we further explored the inhibitory potential of IgG immunoglobulins isolated from ascites samples. Because of our nephelometric analysis, we correlated all ascites samples according to their sodium and IgG content (**Figure 4A**). Considering the results from our functional experiments with dialyzed and heat-inactivated samples in addition, we were able to divide our ascites cohort into four groups: Ascites sample nos. 16 and 21 displayed the highest sodium levels but a low amount of IgG immunoglobulins (upper left quadrant, red). Accordingly, the inhibitory effect of these samples was strongly heat resistant and completely restored after dialysis. In contrast, ascites samples no. 24 and 25 also showed increased sodium content, but also higher amount of IgG immunoglobulins (upper right quadrant, orange), which explained that NK suppression was restored after both dialysis and heat inactivation. The low, if any, NK suppression of benign ascites no. 29 (lower left quadrant, green) with physiological sodium and low IgG content did not change after sample dialysis but improved subsequent to heat inactivation. In contrast, impaired NK cytotoxicity in the presence of ascites sample no. 9 (lower right quadrant, blue) with increased IgG content was completely restored after heat inactivation, exhibiting similar properties to serum samples (purple dots, lower right quadrant).

To further explore the inhibitory potential of immunoglobulins on NK effector function, in the next series of experiments, we studied the impact of IgG in our coculture system. We selected ascites samples containing different combinations of sodium and IgG content (**Figure 4A**, marked in yellow squares) and performed affinity chromatography to isolate the immunoglobulins. We confirmed that the isolation procedure did not lead to any significant loss of immunoglobulins using spectrometry and IgG ELISA (**Figure 4B**). After isolation, the original IgG concentration was restored using ultracentrifugation filters and ascites-derived IgGs were tested in our coculture. Initially, we observed that the isolated immunoglobulins of originally IgG-rich samples (nos. 11 and 13) significantly inhibited NK cell ADCC after 6 h of coculture (**Figure 4C**). The level of ADCC in the presence of IgGs isolated from ascites samples significantly correlated with their concentration (**Figure 4D**). The negative correlation further confirmed the suppressive effects of ascites IgGs.

Following this, we studied the effects of ascites immunoglobulins on NK cell activation and regulatory surface markers CD16 (**Figure 4E**) and DNAM-1 (**Figure 4F**). In both cases, the addition of ascites IgGs significantly prevented activation-induced downregulation of CD16 and DNAM-1 in the NK–tumor cell coculture. Considering that DNAM-1 is downregulated upon contact with its ligands CD112 and CD155, in the following experiment, we studied the impact of ascites IgG on the NK–tumor cell conjugation. Here, we noticed that IgG addition significantly impaired the conjugation of NK cells to target cells (**Figure 4G**).

In summary, the data of this study indicate the existence of two parallel inhibitory mechanisms of antitumoral NK effector functions in malignant ascites. While originally the correlation between ascites IgG and NK cell ADCC in unprocessed ascites was not significant, a negative trend could be noticed (**Figure 4H**). The trend became a significant correlation when NK ADCC was assessed in isolated IgG fraction (**Figure 4I**). This suggested that the IgG immunosupressive effect is masked by much stronger electrolyte-based inhibition and only becomes visible after dialysis or isolation.

## 4 Discussion

One of the main reasons for treatment failure of ovarian cancer and other malignancies is the development of an immunosuppressive tumor microenvironment. Malignant ascites represents a special type of environment since the fluid is enriched by diverse soluble factors such as cytokines, tumor ligands, and metabolites, all of which have inhibitory properties. Despite intense research, the complete understanding of regulatory mechanisms governing immune reactions in the peritoneal cavity are urgently needed, which presents a chance for therapeutical improvements.

In our recently published study, we revealed that electrolyte imbalance is a major inhibitory mechanism in malignant ascites that heavily impaired NK and T-cell effector function (24). Our novel mechanism relied primarily on a hypernatremic environment, which changed calcium flux, gene expression, and recruitment or phosphorylation of signaling proteins in the immune cells. It is important to note that in contrast to a well-studied “high salt” suppressive mechanism (27, 28), in our ascites samples, only sodium concentration was increased, while chloride concentration was below the physiological reference value.

However, in our initial experiments, we noticed that NK cell inhibition in some ascites samples could also be restored by heat inactivation, although imbalance of electrolytes was the major inhibitory factor. Furthermore, dialysis for electrolyte normalization fully reversed inhibitory properties only in samples with high sodium. In samples with low or physiological sodium content, the inhibition was either partially or not reverted at all. Interestingly, there was an inverse correlation between restoring NK functions by heat inactivation and recovery by dialysis. Further experiments with ascites permeates that were protein depleted supported our hypothesis that additive suppression is mediated by other inhibitory, likely proteinaceous component. However, previous published studies had reported inconsistent results regarding heat and protease sensitivity of the inhibitory factors (23, 26, 29), which was likely due to methodical obstacles. For example, chromatography caused the loss of non-proteinaceous fraction, while various precipitation agents could affect the protein stability. The different changes in biological activity of the samples after processing could be the explanation why characterization of the nature of ascites inhibitory component remained contradictory in the past.

Since most of the recent studies primarily focused on ascites cytokine composition and its immune regulatory effect (30, 31), we quantified cytokine concentrations in our ascites samples and correlated them to NK effector function. However, only a correlation in trend was observed for the relationship between the ascites content of IL-8 and IL-10 and NK ADCC. Following this, we assessed their impact on NK activity in our coculture, as both cytokines are considered to impact immune cell function and are highly concentrated in malignant ascites (10). While IL-8 can be stimulatory for many immune cells, other studies reported a largely regulatory and suppressive effect on NK cells (15). Conversely, IL-10, which is one of the suppressive cytokines, is also able to stimulate NK cell effector function by reprogramming their glycolytic metabolism (32). However, in our coculture experiments, cytotoxic NK cell activity was not affected by IL-8 and IL-10 addition. These findings are in line with data from our previous study in which we had observed that the inhibition of immune functions in the presence of ascites is fast-acting and mediated within 5–30 min, which would make cytokine-mediated regulation unlikely (24).

In the following experiments, we confirmed our hypothesis that proteins are responsible for the secondary inhibition in malignant ascites. Furthermore, our studies revealed that both serum proteins and unspecific IgG immunoglobulins in ascites impaired NK cell effector function. To assess this, we added albumin to our coculture, which represents the main component of serum proteins and is also a main part of ascites protein fraction (33, 34). To simulate the presence of irrelevant, unspecific antibodies that are present in ascites, we used the anti-CD20 antibody Rituximab. This therapeutic antibody competed with Cetuximab for binding to NK cell CD16, but could not target CD20-negative ovarian cancer cells in our coculture system. According to our results, both albumin and Rituximab were able to suppress NK cell degranulation and conjugation to target cells, but via distinct mechanisms. Albumin impaired NK activity in the presence of target cells as well as under target-independent conditions during NK cell activation by PMA and Ionomycin. Furthermore, we could show that albumin significantly inhibited the intracellular calcium flux, which was comparable to the sodium-mediated immunosuppressive mechanism (24). This is in accordance with our data showing that healthy donor blood serum also inhibits NK cell activity. Furthermore, using ultracentrifugation, we were also able to show that protein depletion fully abrogated this effect. However, the rationale for immunosuppressive activity of high protein or albumin content in physiological fluids is not understood so far. In a recent study, interaction of Rituximab with human serum albumin was investigated. The study reported that albumin was able to bind Fab- and Fc-antibody domains and CD16 on NK cells, which was associated to reduced NK cell ADCC (35). Our results are also supported by historic studies and observations reporting that serum protein in ascites and even healthy serum proteins impair immune effector functions (36–38). In contrast to the albumin-related effects in our coculture, Rituximab only inhibited cytotoxic NK cell function in the presence of target cells and ADCC-inducing antibody Cetuximab when its concentration greatly exceeded the Cetuximab amount. These data suggest that Rituximab competitively interfered with Cetuximab binding to CD16 on NK cells in our setting. Accordingly, influx of calcium was not affected by Rituximab. Other studies similarly indicated that endogenous serum antibody concentration should be considered prior to intravenous immunotherapy (22, 39). However, to our knowledge, the inhibitory degree of this mechanism has not further been examined in other compartments such as the peritoneal cavity.

For this, based on our mechanistical studies with Rituximab, in our final series of experiments, we examined the impact of immunoglobulins derived from ascites on NK cell activity. In our coculture system, IgG immunoglobulins isolated from ascites samples suppressed NK ADCC and conjugation. Thereby, the degree of inhibition of NK cell ADCC was significantly correlated to the concentration of the isolated immunoglobulins. Furthermore, ascites-derived IgGs prevented downregulation of CD16 and DNAM-1 during NK cell activation, which further augmented our hypothesis of competitive interference in binding CD16 on NK cells.

In summary, this study provides a further understanding of immunoregulatory mechanisms in the tumor microenvironment of advanced ovarian cancer. Here, we report on two distinct protein-related immunosuppressive mechanisms in malignant ascites, which are mediated by ascites-related serum proteins and unspecific immunoglobulins. Importantly, both protein-mediated inhibitory mechanisms are masked in a sodium-enriched environment, but become relevant in biological fluids with physiological or low sodium content. In particular, the competitive interference of endogenous immunoglobulins with therapeutic antibody would be of clinical relevance and should be considered in intraperitoneal antibody-based immunotherapies.

## Data availability statement

The original contributions presented in the study are included in the article/**Supplementary Material**. Further inquiries can be directed to the corresponding author.

## Ethics statement

The studies involving humans were approved by ethics committee, medical faculty of the University Cologne, Cologne, Germany; ethics committee, medical faculty of the University Duisburg-Essen, Essen, Germany. The studies were conducted in accordance with the local legislation and institutional requirements. The participants provided their written informed consent to participate in this study.

## Author contributions

AH: Investigation, Methodology, Conceptualization, Formal analysis, Writing – original draft. SBe: Investigation, Methodology, Writing – review & editing. RK: Resources, Writing – review & editing. SBr: Conceptualization, Funding acquisition, Project administration, Resources, Supervision, Writing – original draft. NM-G: Conceptualization, Funding acquisition, Resources, Supervision, Writing – original draft.
