# Corrigendum: Immunoglobulins and serum proteins impair anti-tumor NK cell effector functions in malignant ascites

**DOI:** 10.3389/fimmu.2024.1420991

**Published:** 2024-04-29

**Authors:** Antonio Hrvat, Sonja Benders, Rainer Kimmig, Sven Brandau, Nina Mallmann-Gottschalk

**Affiliations:** ^1^Experimental and Translational Research, Department of Otorhinolaryngology, University Hospital Essen, Essen, Germany; ^2^Department for Trauma Surgery and Orthopedics, St. Joseph Hospital Kupferdreh, Essen, Germany; ^3^Department of Gynecology and Obstetrics, University Hospital Essen, Essen, Germany; ^4^German Cancer Consortium, Partner Site Essen-Düsseldorf, Essen, Germany; ^5^Department of Gynecology and Obstetrics, University Hospital Cologne, Cologne, Germany

**Keywords:** NK cells, ascites, immunosuppression, ovarian cancer, tumor microenvironment, albumin, antibody, immunoglobulins

In the published article, there was an error in **Figure 2C** as published. The y-axis of **Figure 2C** was mislabeled “Protein [g/dL]” instead of “albumin [g/dL]” by accident. The corrected **Figure 2C** and its caption “(C) Comparison of albumin content in biological fluids. Violin plot illustrating the comparison of albumin content [g/dL] in patient ascites, patient serum, and healthy donor serum”. appear below.

**Figure 2:**
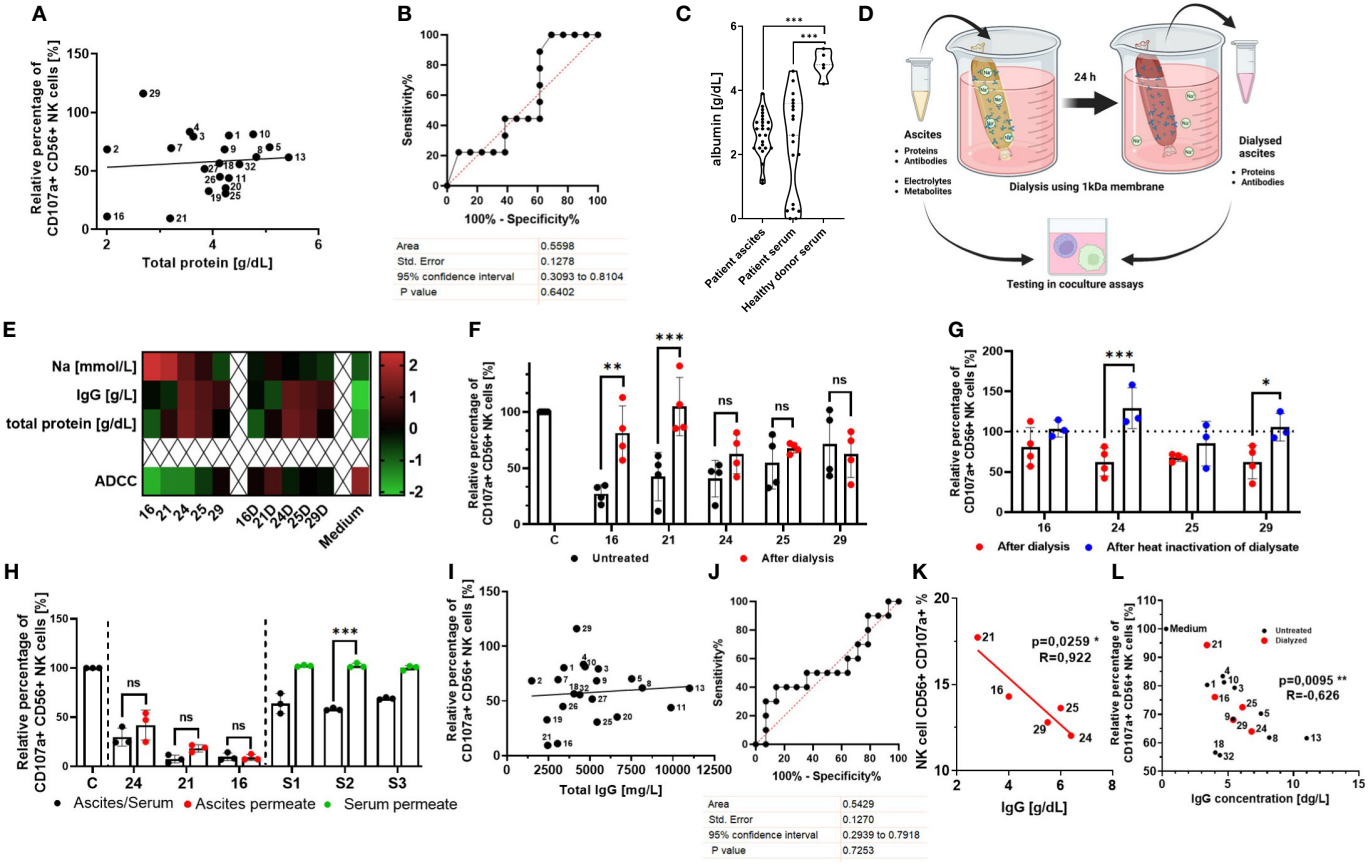
Normalization of imbalanced electrolytes in malignant ascites only partially rescues NK cell ADCC, indicating the existence of an additional inhibitory mechanism. **(A, B)** Relationship between NK ADCC and protein content in ascites samples. **(A)** Pearson correlation shows no significant correlation between NK ADCC [percentage of CD107a-positive NK cells] and protein content [g/dL]. **(B)** ROC (receiver operating characteristic) curve depicting overall protein content as nonsignificant random classifiers. **(C)** Comparison of albumin content in biological fluids. Violin plot illustrating the comparison of albumin content [g/dL] in patient ascites, patient serum, and healthy donor serum. **(D)** Dialysis of ascites samples. Schematic illustration of normalizing ascites electrolyte content via dialysis. Ascites samples were processed in medium overnight using 1-kDa cutoff dialysis tubing to normalize electrolyte composition. Inhibitory effects were assessed in coculture assays. **(E)** Ascites composition before and after dialysis. Heatmap showing concentrations of sodium, IgG, and total protein in selected ascites samples (16, 21, 24, 25 and 26) before (left) and after dialysis (right) (16D,21D,24D,25D,29D) and in culture medium for control (24). **(F)** NK ADCC in the presence of ascites before and after dialysis. Resting NK cells were coincubated in 1:1 ratio with IGROV1 cells and Cetuximab in the presence of untreated (black) or dialyzed ascites (red). After 6 h, expression of CD107a on NK cells was determined by flow cytometry (24). **(G)** NK ADCC in the presence of dialyzed ascites before and after heat inactivation. Resting NK cells were coincubated in 1:1 ratio with IGROV1-cells and Cetuximab with dialyzed ascites (red) and after additional heat inactivation at 56°C for 30 min (blue). **(H)** NK ADCC in the presence of ascites or healthy donor serum after protein depletion. Resting NK cells were coincubated in 1:1 ratio with IGROV1 cells with the addition of Cetuximab in the presence of untreated serum or ascites (black), protein-less ascites permeate (red) and serum permeate (green). After 6 h, expression of CD107a on NK cells was determined by flow cytometry. **(I, J)** Relationship between NK ADCC and IgG-concentration in ascites samples. **(I)** Pearson correlation shows no significant correlation of NK ADCC [percentage of CD107a-positive NK cells] to content of IgG-immunoglobulins [mg/L]. **(J)** ROC (receiver operating characteristic) curve depicting immunoglobulins as nonsignificant random classifiers. **(K, L)** Relationship between NK ADCC and IgG-concentrations in ascites samples with normalized or low sodium content. **(K)** Pearson correlation shows significant correlation of NK ADCC to content of IgG-immunoglobulins [mg/L] in dialyzed ascites samples (red dots). **(L)** Pearson correlation shows significant correlation of NK ADCC to IgG immunoglobulins in dialyzed ascites samples (red dots) and untreated samples with physiological or low sodium content (<145 mM) (black dots). Data are presented as individual values with mean value as center of error bar ± standard deviation. Each datapoint represents one healthy donor. The normalization was done according to normal medium control. For significance testing, ordinary one-way **(C, F, G, H)** ANOVA and Sidak’s post-hoc test, two-tailed Pearson correlation **(A, I, K, L)**, and ROC analysis **(B, J)** were used. *p < 0.05, **p < 0.01, ***p < 0.001.

In the published article, there was an error. Text accidently refers to higher protein concentration instead of albumin concentration. A correction has been made to Results, *3.2 Malignant ascites contains additional inhibitory factors besides imbalanced electrolytes*, second paragraph. This sentence previously stated:

“Furthermore, we observed that healthy donor serum contained higher protein concentrations compared to patient serum and ascites (**Figure 2C**)”.

The corrected sentence appears below:

“Furthermore, we observed that healthy donor serum contained higher albumin concentrations compared to patient serum and ascites (**Figure 2C**)”.

The authors apologize for these errors and state that they do not change the scientific conclusions of the article in any way. The original article has been updated.

