# Supplementary material for: Immunoglobulins and serum proteins impair anti-tumor NK cell effector functions in malignant ascites
Source: Front Immunol. 2024 Apr 5;15:1360615. doi: 10.3389/fimmu.2024.1360615 (PMC11026578; doi:10.3389/fimmu.2024.1360615)
Supplement: Supplementary file 1 [file DataSheet_1.docx]

# Supplementary Material


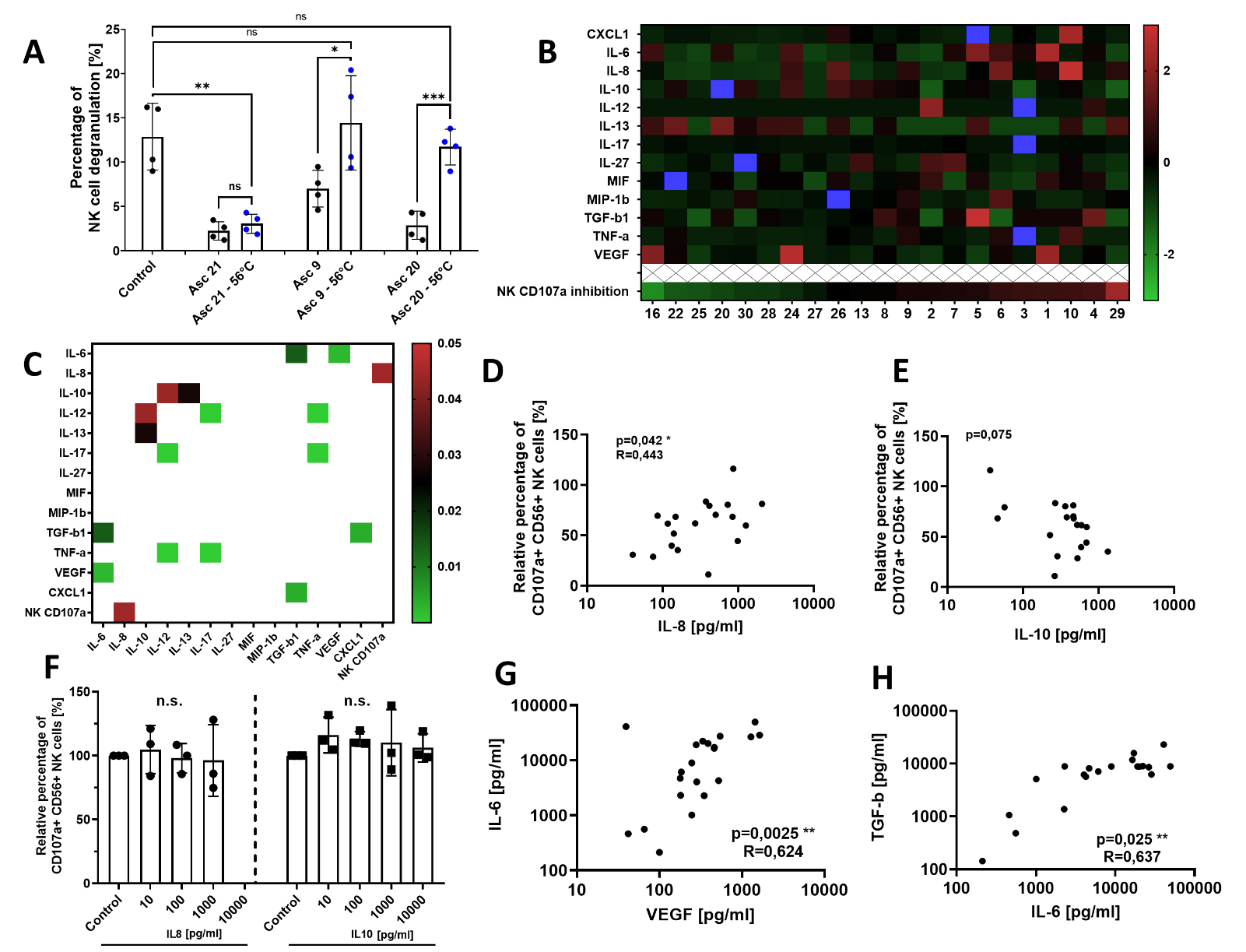


**Supplementary figure S1. Ascites cytokines are not correlated with suppressive effects on NK cell degranulation**. **(A)** **Heat inactivation of ascites partially restores NK ADCC in only some ascites samples**. NK ADCC in presence of heat inactivated ascites. Resting NK cells were coincubated in 1:1 ratio with IGROV1-cells and Cetuximab with dialyzed ascites (black), which was heat inactivated at 56°C for 30 minutes (blue). **(B)** **Quantification of ascites cytokines**. Cytokines of ascites samples were determined by ELISA. Presented in the heatmap are calculated z-score values for each component. **(C-F)** **Correlation between quantified ascites cytokines and NK effector function.** **(C)** Heatmap showing significant correlations (p<0,05) between ascites cytokines and NK ADCC. Correlation between NK ADCC and concentrations of **(D) IL-8** or **(E) IL-10** in ascites samples**.** **(F) NK ADCC in presence of IL-8 and IL-10.** Resting NK cells were coincubated with IGROV1 cells (1:1 ratio) and addition of Cetuximab in medium supplemented with different concentrations of IL-8 (left) or IL-10 (right). Correlation between cytokines: **(G) IL-6 and VEGF** and **(H) TGF-β and IL-6**. Data are presented as individual values with mean value as center of error bar ± standard deviation. For significance testing unpaired t-test (S1. A), two-tailed Pearson correlation (S1.C, D, E, G and E) and ordinary one-way ANOVA with Dunnett posthoc (S1. F) were used. ns (non-significant), *(p<0.05), **(p<0.01), ***(p<0.001).


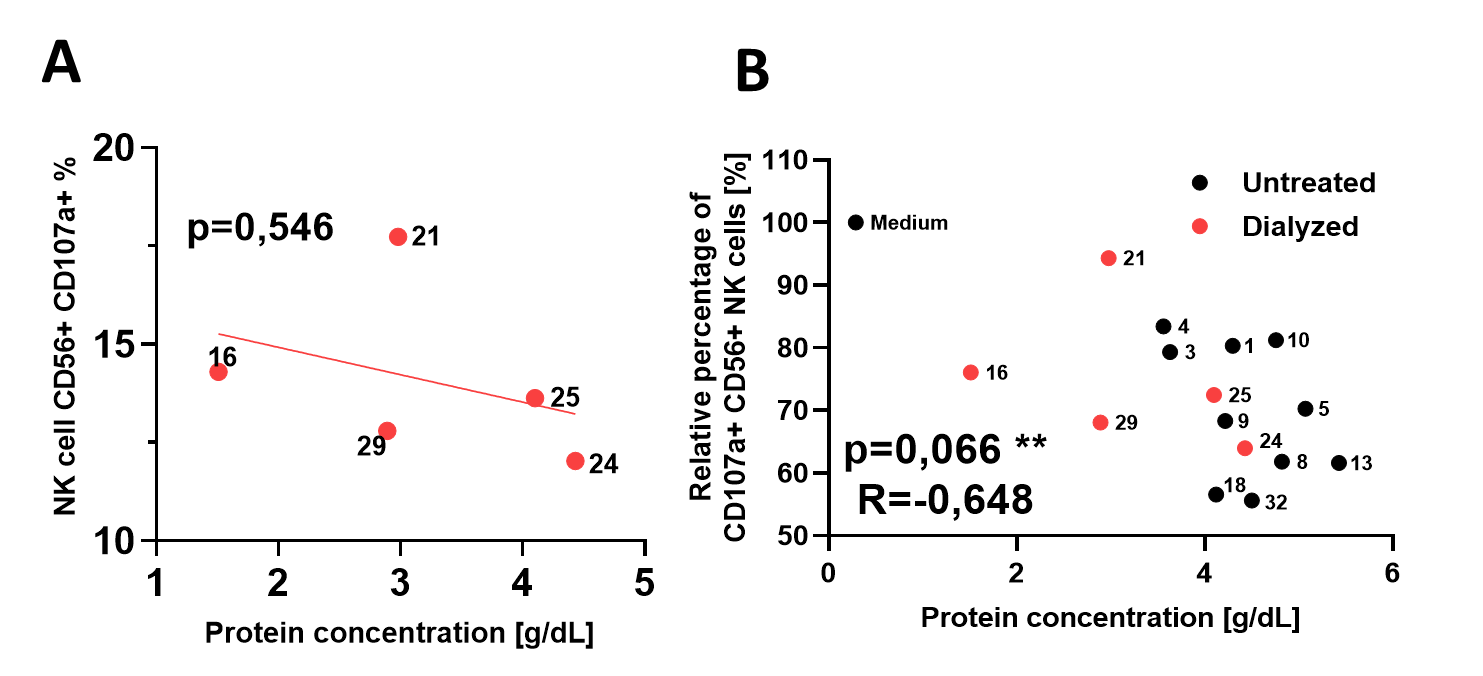


**Supplementary figure S2. (A-B) Relationship between NK cell ADCC and proteins in ascites samples with physiological or normalized sodium content** **(A)** Pearson correlation shows no significant correlation of NK ADCC to protein content in dialyzed ascites samples (red). **(B)** Pearson correlation shows no significant correlation of NK ADCC to protein content in dialyzed ascites samples (red) and untreated (black) samples with physiological or low sodium content (<145 mM). Each datapoint represents one healthy donor. For significance testing two-tailed Pearson correlation (S2. A and B) was performed. ns (non-significant), ** (p<0.01).
